# Cross-sectional dietary deficiencies among a prison population in Papua New Guinea

**DOI:** 10.1186/1472-698X-13-21

**Published:** 2013-04-22

**Authors:** Camilla Gould, Benoit Tousignant, Garry Brian, Robert McKay, Rosalind Gibson, Karl Bailey, Bernard J Venn

**Affiliations:** 1 Department of Human Nutrition, University of Otago, P.O. Box 56, Dunedin 9054, New Zealand; 2 The Fred Hollows Foundation New Zealand, Private Bag 99909, Newmarket, Auckland 1149, New Zealand; 3 Faculty of Health Sciences, Divine Word University, Madang, Papua New Guinea, 1 Nabasa Road, Madang, Madang Province 511, Papua New Guinea; 4 Dunedin School of Medicine, University of Otago, P.O. Box 913, Dunedin 9054, New Zealand

**Keywords:** Prisoner, Prison, Rations, Diet, Nutrition, Deficiency

## Abstract

**Background:**

To investigate the dietary adequacy of prisoners of Beon Prison, Madang, Papua New Guinea in response to a report of possible nutritional deficiency.

**Methods:**

We undertook an observational, cross-sectional study. All 254 male inmates (May 2010) were eligible to answer a validated interview-based questionnaire; to have a comprehensive dietary assessment; and to provide blood for biochemical analysis (α-tocopherol, β-carotene, lutein, thiamin, riboflavin, niacin, folate, homocysteine, zinc, ferritin, and vitamins A, B_12_ and C). Prison guards were invited to participate as a comparison group.

**Results:**

148 male prisoners (58.3%) and 13 male prison guards participated. Prison rations consisted of white rice fortified with thiamin, niacin, and iron, tinned tuna, tinned corned beef, water crackers, and black tea, with occasional intakes of fruit and vegetables. Some prisoners received supplementary food from weekend visitors. From assessment of the prisoners dietary data, median intakes of calcium (137 mg), potassium (677 mg), magnesium (182 mg), riboflavin (0.308 mg), vitamin A (54.1 μg), vitamin E (1.68 mg), vitamin C (5.7 mg) and folate (76.4 μg) were found to be below estimated average requirements (EAR).

Following are the prisoners median (P_25_, P_75_) concentration of circulating nutrients and the percentage of prisoners with levels below normal reference ranges or recognized cut-off values: serum retinol 0.73 (0.40, 1.21) μmol/L, 46% below 0.7 μmol/L; plasma folate 2.0 (1.4, 2.6) nmol/L, 98% below 6.8 nmol/L; plasma vitamin C 6.3 (1.0, 19.3) μmol/L, 64% below 11.4 μmol/L; serum zinc 9.9 (8.8, 11.1) μmol/L, 66% below 10.7 μmol/L. Guards had diets with a higher dietary diversity that were associated with greater intakes of nutrients and biomarker concentrations.

**Conclusions:**

The prisoners diets are likely lacking in several micronutrients and recommendations for dietary change have been made to the prison authorities. Ongoing vigilance is required in prisons to ensure the basic human right of access to a nutritionally adequate diet is being observed.

## Background

Prisoners incarcerated in developing countries have been shown to be vulnerable to dietary deficiencies. Outbreaks of scurvy [1], vitamin A deficiency disorders [2] and beriberi [3] have been documented in various African prisons. The local ophthalmologist in Madang, Papua New Guinea, became aware of adult prisoners developing vision loss during incarceration at the local correctional facility, Beon Prison. For several of the prisoners this was attributed to optic neuropathy, a condition that may lead to permanent optic nerve dysfunction and blindness although it is rarely present in the general population [4]. The prisoners presenting to the local eye clinic described a diet with little fruit and vegetables, possibly indicative of an inadequate intake of some nutrients. Indeed, nutritional deficiencies and toxic substances have been associated with optic neuropathy [5,6]. In response to the report, and in order to provide evidence-based advice to prison authorities, an ophthalmic and nutritional survey was undertaken to identify the prevalence of optic neuropathy and to investigate potential nutritional and toxic correlates of the disease. As a result of this investigation, optic neuropathy with a prevalence estimate of 10.4% among prisoners was found to be associated with length of incarceration, age, and low blood folate concentrations [7]. Some prisoners exhibited xerophthalmia associated with vitamin A deficiency [8]. In addition to investigating the prevalence and risk factors associated with optic neuropathy and visual loss, a broader dietary assessment was undertaken to determine whether the inmates of Beon Prison were at risk of other nutrient deficiencies. Here we report on the adequacy of the diets of the prisoners in terms of nutrient intakes and blood biochemistry.

## Methods

In May 2010 there were 254 adult male (≥18y) prisoners at Beon Prison, Madang, Papua New Guinea. Adult male prison guards were also invited to participate in the study as a comparison group. The purpose of the study was explained to all adult prisoners and guards and assurances were given regarding confidentiality of the data. Communications with potential participants were conducted in Papua New Guinean Pidgin (*Tok pisin*). Care was taken during the investigation to minimize any potential repercussions for participants from both fellow inmates and authorities.

### Dietary and lifestyle questionnaire

An interview-based questionnaire was designed to elicit demographic, socioeconomic, dietary and lifestyle information. Health workers and student health extension officers administered the questionnaire. In addition to dietary intake, questions were asked about smoking and the chewing of betel nut. Dietary intake was assessed using a single interactive 24-hour recall using multiple-pass interviewing [9]. Prompts were given based on prison rations and locally available foods brought into the prison by visitors at weekends. Graduated food models, measuring cups, spoons, and prison cups and plates were made available to prisoners and guards to aid with portion estimation. In addition, thirty prison lunches and dinners were weighed over three days using Salter Electronic Kitchen Scales (Model 1035, Salter Housewares Ltd., Kent, UK) accurate to ± 2 grams. The guards also completed a food frequency questionnaire (FFQ). The nutrient composition of foods were derived primarily from the nutrition information panel or from the Pacific Islands Food Composition Table [10]. For foods not listed in the Pacific Islands dataset, the Australian [11] or the New Zealand [12] food composition tables were used. The nutrient contents of the foods were combined with the intake estimates to yield median (IQR) daily intakes of 26 nutrients. The average energy requirements of prisoners and guards were calculated using individual energy requirements based on body mass and an assumed sedentary physical activity level of 1.4 in accordance with WHO criteria [13].

A fasting venous blood sample was drawn from participants into two evacuated tubes; a trace-element and anticoagulant-free tube, and an EDTA treated vacutainer tube (Becton Dickinson Rutherford, NJ, USA). The tubes were immediately refrigerated and kept in the dark. Within 30 minutes of collection, aliquots of whole blood were taken from the EDTA tubes; the tubes were then centrifuged and aliquots of plasma removed. One plasma aliquot was treated with metaphosphoric acid in preparation for vitamin C analysis. A sample of the red blood cells were washed and stored for thiamin analysis. One hour after collection, blood in the trace-element free tubes was centrifuged, haematocrit was determined, and serum was aliquoted into trace-element free polyethylene storage tubes. All blood sample preparation was conducted with minimal exposure to light and plasma samples for carotenoid and riboflavin analysis were wrapped in aluminium foil. Samples were stored at −80°C, freighted frozen on dry ice to New Zealand, and stored at −80°C until analysed.

Biochemical tests were generally undertaken according to published methods or using manufacturer’s kits as follows: serum retinol, α-tocopherol, β-carotene and lutein by high performance liquid chromatography (HPLC) [14]; plasma and whole blood folate by microbiological assay [15]; plasma vitamin C by fluorometric assay [16]; plasma selenium using electrothermal atomic absorption spectrophotometry [17]; plasma total homocysteine (tHCy) by HPLC [18]; and serum zinc using atomic absorption spectrophotometry [19]. Manufacturer’s kits were used for serum ferritin, vitamin B_12_, cholesterol and creatinine (Roche Diagnostics GmbH, Mannheim, Germany), serum α-1-glycoprotein (Randox Laboratories Ltd, Crumlin, UK), and whole blood riboflavin (Chromsystems Instruments & Chemicals GmbH, München, Germany). Red blood cell thiamin pyrophosphate was measured by HPLC using an in-house method developed by Canterbury Health Laboratories (Christchurch, New Zealand). Accuracy and precision were monitored and maintained using commercial controls for α-tocopherol, β-carotene, retinol, lutein, selenium, vitamin B_12_, riboflavin, tHcy, cholesterol and creatinine. A reference standard (National Institute ST) was used for folate. Pooled plasma was used to check precision of the thiamin and vitamin C assays.

Differences in the demographic characteristics of prisoners and guards were tested using the Fisher’s exact test for categorical variables and the Student’s t-test for continuous variables. Differences between nutrient intakes and biomarkers of prisoners and guards were tested using the median test. Analyses were performed using Intercooled Stata 9.2 (Stata Corporation, College Station, TX, USA). Statistical significance was accepted at *P*<0.05.

Ethics approval was granted both by the institutional ethics review board for the local university (Divine Word University Research Ethics Committee) and by the Medical Research Advisory Committee of Papua New Guinea (MRAC 10.16). All participants gave informed written consent for participation. The tenets of the Helsinki Declaration were observed.

### Role of the funding source

The New Zealand Agency for International Development (NZAID) financially supported the design, implementation and analysis of this study through the Kaihono hei Oranga Hapori o te Ao partnerships for International Community Development scheme (KOHA-PICD), but had no input into or control over content or commentary. The Department of Human Nutrition of the University of Otago provided funding for the design and implementation of the dietary and blood assessment comprising dietary data collection, input and analysis and the on-site processing and laboratory analysis of the blood samples. The Fred Hollows Foundation New Zealand provided funding for the design and implementation of clinical data collection, general study administration, data analysis and write-up.

## Results

Consent and baseline data were obtained from 148 prisoners and 13 guards. The baseline characteristics of the prisoners and guards are presented in Table 1. All study participants were of Melanesian ethnicity. Prisoners had a significantly lower body weight and BMI, and were younger than the guards. According to the WHO (2000) classification, 5% of the prisoners were underweight (BMI<18.5), 81% had a BMI within the normal range (BMI ≥18.5-24.9), and 15% were overweight (BMI ≥25-29.9) [20]. Fifty-seven prisoners (39%) and 4 guards reported weight loss within the previous 3–6 months. The majority of the prisoners identified themselves as current tobacco smokers despite access to tobacco in the prison being restricted; the number of cigarettes smoked was generally less than 10 per day. Few prisoners reported consuming alcohol in prison, although the majority had been consumers before incarceration.

**Table 1:** Descriptive characteristics of male prisoners and guards at Beon prison

**Characteristic**	**Prisoners n=148**^**a**^	**Guards n=13**^**a**^	***P ***^**b**^
	**Mean (SD)**	**Mean (SD)**	
Age (years)	31.0 (7.93)	47.3 (10.2)	<0.001
Height (cm)	164 (6.83)	167 (4.27)	0.112
Weight (kg)	60.6 (6.92)	75.8 (14.1)	<0.001
BMI (kg/m^2^)	22.5 (2.25)	27.1 (5.22)	<0.001
Time incarceration at Beon (months)	24.7 (27.9)	-	
	**n (%)**	**n (%)**	
Smoking status			
Current	103 (71)	3 (23)	<0.001
Past	31 (21)	0 (0)	
Never	12 (8.2)	10 (77)	
Tobacco use			
None	43 (29)	9 (75)	
<1 cigarette/day	48 (33)	0 (0)	0.001
1-10 cigarettes/day	46 (32)	1 (8.3)	
≥10 cigarettes/day	9 (6.2)	2 (17)	
Alcohol status			
Current	19 (13)	8 (62)	<0.001
Past	115 (79)	0 (0)	
Never	12 (8.2)	5 (38)	
Alcohol consumption			
None	128 (90)	5 (38)	
<1 unit/day	1 (0.7)	0 (0)	<0.001
1-2 units/day	2 (1.4)	2 (15)	
≥3 units/day	12 (8.4)	6 (46)	

^a^ Not all data are complete.

^b^*P* value generated from Student’s t-test (parametric data) or Fisher’s exact test (non-parametric data).

^c^ Unit of alcohol = 1 bottle/can beer, 125 mL home-made alcohol, 1 glass of wine or 10 mL spirits.

The prison rations consisted of water-crackers and black tea for breakfast; and white rice topped with tinned corned beef or tuna for the midday and evening meals. There were reports of small amounts of fruit and vegetables being supplied sporadically. Water was available throughout the day. Another source of food for some prisoners was food brought in by weekend visitors. This food had to be eaten in the visiting area and it could not be shared with other prisoners. Most prisoners reported consuming fruit and vegetables rarely or never (66%), and 91% reported consumption of these foods less than once per week. In contrast, most guards reported consuming green vegetables, rice and coconut milk on a daily basis with fruit, fish, meat and nuts consumed 1–3 times/week.

Dietary data based on 24-hr recalls were elicited from 148 prisoners and 9 guards. The mean (SD) portion size of rice estimated by the prisoners was 418 g (132); this compares well with the mean of 423 g (62) obtained from weighing 40 rice servings. There was a tendency for the prisoners to underestimate the portion of corned beef and tuna by approximately one-third. The prisoners estimated a topping of corned beef and tuna to be 40 g (27.1) and 41 g (19.6), respectively, whereas the weighed amounts were 58 g (5.7) and 61 g (5.9).

The nutrient intakes generated from the dietary recalls are presented in Table 2. When compared with the guards, prisoners had significantly lower intakes of fat, saturated fat, vitamin A, riboflavin, folate, vitamin C, vitamin E, potassium and calcium. The median energy intake of the prisoners was 7430 kJ (P_25_ 6170, P_75_ 8600) compared with an estimated requirement based on FAO calculations of 7618 kJ (P_25_ 7952, P_75_ 8285). For the guards the estimated intake was 8360 kJ (P_25_ 6990, P_75_ 9150) with a requirement of 8610 kJ (P_25_ 8142, P_75_ 8809) [13].

**Table 2:** Median daily nutritional intake of male prisoners and guards based on 24-hour dietary recall data and gift recall data

	**Prisoners n=148**	**Guards n=9**	***P***^**1**^
	**Median (P**_**25**_**, P**_**75**_**)**	**Median (P**_**25**_**, P**_**75**_**)**	
Energy (kJ)	7430 (6170, 8600)	8360 (6990, 9150)	NA^2^
Protein (g)	50.5 (41.2, 59.6)	73.3 (46.2, 78.2)	NA^2^
Total fat (% energy)	13.0 (11.6, 15.0)	30.1 (26.8, 42.0)	0.002*
Saturated fat (% energy)	7.15 (6.07, 8.34)	13.6 (10.2, 16.9)	0.015*
Cholesterol (mg)	45.9 (29.9, 57.9)	109.0 (47.0, 158.0)	0.083
Carbohydrates (% energy)	76.4 (73.7, 82.0)	57.7 (47.1, 62.8)	0.002*
Total dietary fibre (g)	9.80 (8.08, 11.8)	16.9 (15.5, 17.4)	0.083
Total vitamin A (μg)	54.1 (14.8, 104)	666 (248, 857)	0.014*
ß-carotene equiv (μg)	556 (27.6, 1100)	6620 (1035, 7710)	0.069
Retinol (μg)	12.4 (7.66, 16.6)	129 (52, 159)	0.083
Thiamin (mg)	1.09 (0.875, 1.30)	0.97 (0.75, 1.10)	0.372
Riboflavin (mg)	0.308 (0.242, 0.383)	0.812 (0.614, 1.09)	0.001*
Niacin equiv. (mg)	23.7 (19.5, 28.2)	22.8 (21.5, 27.1)	0.746
Vitamin B_6_ (mg)	1.57 (1.02, 1.85)	1.58 (1.13, 1.87)	0.717
Vitamin B_12_ (μg)	2.83 (1.49, 3.11)	3.06 (2.11, 4.59)	0.495
Folate (μg)	76.4 (61.3, 93.1)	238 (108, 263)	0.015*
Vitamin C (mg)	5.7 (1.3, 10.2)	85.1 (29.5, 105.0)	0.012*
Vitamin E (mg)	1.68 (1.15, 2.30)	7.69 (4.55, 9.84)	0.015*
Sodium (mg)	1560 (1230, 1920)	1710 (1350, 2340)	0.717
Magnesium (mg)	182 (150, 223)	290 (233, 328)	0.083
Potassium (mg)	677 (512, 870)	1970 (1560, 2844)	0.002*
Calcium (mg)	137 (107, 202)	382 (287, 496)	0.015*
Iron (mg)	12.7 (10.3, 15.3)	13.7 (10.3, 16.3)	0.717
Zinc (mg)	8.71 (6.78, 10.4)	7.92 (4.90, 9.72)	0.746

^1^ Generated from Median test.

^2^ NA – Not Applicable; Prisoners and guards have different requirements based on body mass.

* Significant *P*-value p<0.05.

**Figure 1:**
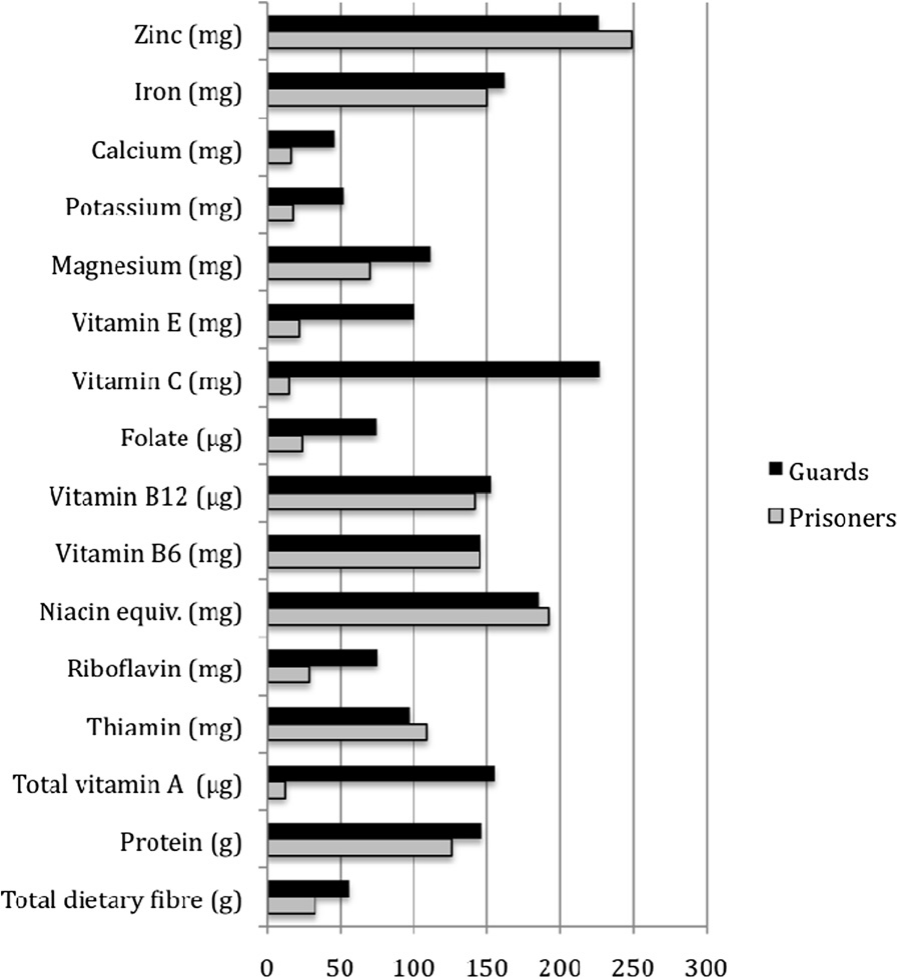
**Median nutrient intakes of male prisoners and guards as percentages of EARs**^**a**^**. **^a^ The following estimated average requirement (EAR) values were used for calculations: total vitamin A, 429 µg; thiamin, 1.0 mg; riboflavin, 1.1 mg; niacin equiv., 12 mg; vitamin B6, 1.1 mg; vitamin B12, 2.0 µg; folate, 320 µg; vitamin C, 38 mg; vitamin E, 7.7 mg; magnesium, 260 mg (recommended nutrient intake); calcium, 830 mg; iron, 8.5 mg; zinc 3.5 mg; (zinc was taken as being highly bioavailable, iron was taken as being 12% bioavailable) [21]; total dietary fibre, 30 g; potassium, 3800 mg (adequate intake) [22]; protein, 40 g (recommended dietary intake) [23].

The median intakes of nutrients expressed as a proportion of the estimated average requirement (EAR) are shown in Figure 1. The median intake of the prisoners was close to or exceeded the EAR for protein, zinc, iron, vitamin B_12_ and B_6_, niacin and thiamin. Less than 25% of the prisoners met the EAR for vitamin A, folate, vitamin C, vitamin E, potassium and calcium assuming their intake from the 24-hour recall was representative of their usual intake. The intakes of these marginal nutrients were somewhat enhanced in the diets of seven prisoners who received food regularly once a week from visitors. On average, these prisoners consumed an extra 148 μg vitamin A (35% EAR), 0.12 mg riboflavin (11% EAR), 31.5 μg folate (10% EAR), 18.3 mg vitamin C (49% EAR) and 451 mg potassium (10% EAR) when compared with prisoners who did not receive food gifts. As depicted in Figure 1, the guards’ intake was closer to the EAR for the majority of nutrients compared with the prisoners’.

Fasting blood samples were collected from 139 prisoners and 9 guards. As shown in Table 3, over half of the prisoners had biomarker concentrations below recommended cut-offs for retinol, vitamin C and zinc. Nearly all prisoners had deficient plasma folate concentrations. Most had red blood cell folate concentrations indicative of depletion whilst approximately one-third were deficient. Shorter-term prisoners (<18 weeks incarceration) were found to have significantly higher red blood cell folate concentrations (390 nmol/L: 95% CI 370, 410) compared with longer-term prisoners (260 nmol/L: 95% CI 240, 280) (p<0.001). Most prisoners were hyperhomocysteinemic, with approximately one-third having severe hyperhomocysteinemia (50-500 μmol/L). Several of the guards also had low folate and high homocysteine concentrations. Biomarkers of α-tocopherol, thiamin, vitamin B_12_ and selenium were largely within the normal ranges for both prisoners and guards. The guards had a significantly lower red cell thiamin pyrophosphate concentration than the prisoners (p<0.05).

**Table 3:** **Median (P**_**25**_**, P**_**75**_**) blood indices of nutrient status in male prisoners and guards (all tests undertaken with serum unless otherwise stated)**

	**Prisoners (n=139**^**a**^**)**	**Guards (n=9**^**a**^**)**	***P***	**Cut-off**^**b**^	**Prisoners < or > cut-off n (%)**
Retinol (μmol/L)	0.73 (0.40, 1.21)	1.36 (0.96, 1.67)	0.016	<1.05 marginal	90 (65)
				<0.70 deficient	63 (46)
ß-Carotene (μmol/L)	<0.06 (<0.06, 0.09)	0.25 (0.15, 0.36)	0.000		
α-Tocopherol (μmol/L)	15.5 (12.5, 18.3)	23.2 (20.2, 27.0)	0.002	<11.6 deficienct	25 (18)
Cholesterol (mmol/L)	4.4 (3.8, 5.1)	5.5 (4.6, 7.0ß)	0.016	>5.2 high	27 (20) ^c^
α-Tocopherol: total cholesterol ratio (μmol/ mmol)	3.43 (2.96, 3.81)	3.67 (4.04, 5.09)	0.016	<2.2 inadequate	0 (0)
Lutein (μmol/L)	1.58 (1.13, 2.40)	5.48 (4.34, 6.91)	0.002		
Plasma vitamin C (μmol/L)	6.3 (1.0, 19.3)	48.5 (17.2, 59.4)	0.002	<11.4 deficienct	87 (64)
Red cell thiamin pyrophosphate (nmol/L)	258 (215, 307)	167 (133, 200)	0.016	<140 risk of suboptimal status	1 (1)
Plasma riboflavin (nmol/L)	221 (164, 282)	333 (255, 377)	0.013	<155 deficienct	25 (18)
Plasma folate (nmol/L)	2.0 (1.35, 2.6)	2.8 (1.6, 4.0)	0.246	<6.8 negative balance	134 (98)
Red blood cell folate (nmol/L)	270 (180, 348)	380 (262, 492)	0.085 0.168	<363 depletion	105 (80)
				<227 anemia	46 (35)
Vitamin B_12_ (pmol/L)	465 (382, 564)	340 (289, 440)	0.016	< 130 risk of deficiency	0 (0)
Holotranscobalamin II (pmol/L)	76 (62.5, 93)	64 (53, 82.5)	0.302	<23 deficienct	0 (0)
Plasma homocysteine (μmol/L)	36 (18, 58)	16 (10, 56)	0.388	>15 moderate hhcy	108 (79)^c^
				>25 intermediate	92 (68)^c^
				50-500 severe	48 (35) ^c^
Selenium (μmol/L)	1.59 (1.48, 1.75)	1.55 (1.49, 1.88)	0.333	<1.27 inhibited GSHPx activity	0 (0)
Zinc (μmol/L)	9.9 (8.8, 11.1)	10.2 (9.0, 10.8)	0.974	<10.7	90 (65.7)
Ferritin (μg/L)	73 (40, 120)	90 (77, 141)	0.2063	<15 depleted stores	10 (7.6)
α-1 acid glycoprotein (mg/dL)	64 (54, 73)	56 (55, 70)	0.4885	>120 subclinical infection	1 (0.76)

^a^ Not all data are complete.

^b^ Cut-offs used to define risk of micronutrient deficiencies were obtained from the following sources: serum retinol [24]; serum vitamin C [25]; serum α-tocopherol [26]; serum α-tocopherol: total cholesterol ratio [25]; red cell thiamin [27]; serum vitamin B_12_[28]; serum holotranscobalamin II [26]; plasma FAD (Canterbury Laboratories), haematocrit [29], plasma folate [30]; plasma homocysteine [31]; serum selenium [32]; total serum cholesterol [33]; serum iron [21]; serum zinc [34]; serum α-1 acid glycoprotein [35].

^c^ n(%) above cut-off.

* Significant *P*-value p<0.05.

## Discussion

Our investigation highlights the dietary deficiencies of prisoners incarcerated at Beon Prison. Xerophthalmia in the prisoners was found to be associated with a low intake of vitamin A [8] and optic neuropathy with low folate status [7]. The extremely low folate concentrations found would render the prisoners at high risk of megaloblastic anaemia [30]. Additionally, high homocysteine concentrations are associated with an increased risk of arteriosclerosis [36]. Most of the prisoners had inadequate vitamin C intakes and 64% had deficient plasma vitamin C concentrations placing them at risk of scurvy.

The guards were consuming a wider variety of foods associated with better biochemical indices for several nutrients. Our ability to compare the diets of the prisoners to that of local residents as a means of placing the prison rations in context was limited to just a few guards and in this regard, a larger sample of non-prisoners would have been beneficial. Nevertheless, the prisoners’ mean intake of vitamin A, riboflavin, folate, vitamin C, vitamin E and potassium were significantly below that of the guards suggesting an adverse disparity in the diets of the prisoners compared with the outside population. Indeed, the guards report a regular consumption of green vegetables, fruit and nuts, food items that were rarely available to the prisoners. Both prisoners and guards had low calcium intakes, almost certainly a consequence of a lack of dairy products being consumed with both groups being at risk of osteomalacia. Red cell thiamin was higher in the prisoners compared with the guards, presumably as a consequence of the prisoner’s rice being fortified with thiamin. The serum concentration of selenium was above cut-off concentrations below which the activity of selenium-dependent enzymes are compromised [32].

Although the dietary intakes are based only on one recall, we have some reassurance regarding the accuracy of the estimates because biomarker concentrations in the prisoners were generally congruous with the nutrient intake data. Low intakes of folate, vitamin C, and vitamin A were reflected in low blood concentrations of these micronutrients and an adequate intake of thiamin and vitamin B_12_ corresponded to sufficient concentrations of thiamin, vitamin B_12_ and holotranscobalamin II (a vitamin B_12_ metabolite) in the blood. The estimated riboflavin intake of the prisoners of 0.3 mg/d was well below the EAR and considerably less than 0.5-0.6 mg/d, the amount considered essential to avoid clinical signs of deficiency [37]. However, despite these low intakes, most prisoners had plasma riboflavin concentrations above the cut-off for deficiency of this metabolite. Given that the intake and biochemical data are incongruous, it is possible that the prison rations contained more riboflavin than indicated from the food composition tables. The estimated vitamin E intake was just one-sixth of the EAR whereas the serum measures (α-tocopherol and the ratio of α-tocopherol/cholesterol) were for the majority of prisoners above the cut-off values. This mismatch is not necessarily inconsistent because circulating α-tocopherol concentrations have been found not to correlate with dietary intake of vitamin E [38]. The energy intakes of the prisoners were somewhat less than calculated requirements although the mean BMI of the prisoners was in the healthy range. The absence of beriberi in Beon Prison is almost certainly due to the fortification of white rice with thiamin. Care should be taken if the supply of rice changes from the current fortified rice to an unfortified source as this may lead to thiamin deficiency.

The provision of a nutritionally adequate diet is a basic human right [39] and acknowledgement of this right is contained in the constitutional documents of Papua New Guinea [40]. The inadequacy of the prison diet is placing the prisoners at risk of acute and chronic nutrient deficiency diseases. The prison rations are supplemented in some prisoners with food brought in by weekend visitors, but not all prisoners receive visitors and the nutrient contribution of the additional food to the composition of the overall diet is small. The prison diet is likely chosen because of ease of storage, preparation and availability of food items. However, the right to adequate food is not being met by the current prison feeding regimen. With the prior consent of the prisoners, the prison authorities were notified of those prisoners diagnosed as having xerophthalmia and advised to supply them with vitamin A supplements. More generally, recommendations have been made to the authorities to procure multivitamin and mineral supplements and in the longer term, to improve the diet quality of the prisoners by the inclusion of fruit, vegetables and milk powder. Locally available foods such as avacado, banana, papaya, green leafy vegetables including aibika and broccoli, and orange colored sweet potato (kaukau) will provide several of the deficient nutrients including folate, vitamin C, fibre and provitamin A carotenoids. Milk powder available in local supermarkets would be a good source of calcium and the provision of three cups of milk per day, and adding milk powder to tea, would substantially improve calcium intakes. Calcium would also be provided by the consumption of small whole fish with soft bones, although the availability of this on a regular basis may be difficult.

## Conclusions

The prison rations were lacking in variety resulting in an inadequate intake of several essential nutrients to prisoners. Testing of blood samples confirmed that dietary inadequacy was reflected in biochemical indicators. From a clinical eye examination, the nutrition related diseases xerophthalmia and optic neuropathy were identified. Elsewhere, severe malnutrition has been documented in prisoners in Zimbabwe [41], Democratic Republic of Congo, as well as vitamin A deficiency in Kenya [2] and beriberi in the Cote d’Ivoire [3,42]. Clearly, ongoing vigilance of prisoners’ diets is required.

## Competing interests

The authors declared that they have no competing interest.

## Authors’ contributions

CG, BV, RG, BT and GB designed the study. CG, BV, BT and RM conducted the on-site investigation. CG and KB conducted the laboratory blood analysis. CG analysed the dietary data. Statistical analysis was undertaken by CG and BV, and data interpretation by CG, BV, RG, BT and GB. The initial draft manuscript was prepared by CG, BV, RG, BT and GB with all authors involved in editing subsequent drafts. All authors read and approved the final manuscript.

## Pre-publication history

The pre-publication history for this paper can be accessed here:

http://www.biomedcentral.com/1472-698X/13/21/prepub
